# SALL4, a Stem Cell Factor, Affects the Side Population by Regulation of the ATP-Binding Cassette Drug Transport Genes

**DOI:** 10.1371/journal.pone.0018372

**Published:** 2011-04-19

**Authors:** Ha-Won Jeong, Wei Cui, Youyang Yang, Jiayun Lu, Jie He, Ailing Li, David Song, Ye Guo, Bee H. Liu, Li Chai

**Affiliations:** 1 The Department of Pathology, Brigham and Women's Hospital, Harvard Medical School, Boston, Massachusetts, United States of America; 2 Department of Clinical Laboratory, Chinese Academy of Medical Sciences, Peking Union Medical College Hospital, Beijing, China; 3 Centre for Life Sciences, Cancer Science Institute of Singapore, National University of Singapore, Singapore, Singapore; University of Barcelona, Spain

## Abstract

Our previous work shows that the stem cell factor SALL4 plays a central role in embryonic and leukemic stem cells. In this study, we report that SALL4 expression was higher in drug resistant primary acute myeloid leukemic patients than those from drug-responsive cases. In addition, while overexpression of SALL4 led to drug resistance in cell lines, cells with decreased SALL4 expression were more sensitive to drug treatments than the parental cells. This led to our investigation of the implication of SALL4 in drug resistance and its role in side population (SP) cancer stem cells. SALL4 expression was higher in SP cells compared to non-SP cells by 2–4 fold in various malignant hematopoietic cell lines. Knocking down of SALL4 in isolated SP cells resulted in a reduction of SP cells, indicating that SALL4 is required for their self-renewal. The SP phenotype is known to be mediated by members of the ATP-binding cassette (ABC) drug transport protein family, such as ABCG2 and ABCA3. Using chromatin-immunoprecipitation (ChIP), quantitative reverse transcription polymerase chain reaction (qRT-PCR) and electrophoretic mobility shift assay(EMSA), we demonstrated that SALL4 was able to bind to the promoter region of ABCA3 and activate its expression while regulating the expression of ABCG2 indirectly. Furthermore, SALL4 expression was positively correlated to those of ABCG2 and ABCA3 in primary leukemic patient samples. Taken together, our results suggest a novel role for SALL4 in drug sensitivity, at least in part through the maintenance of SP cells, and therefore may be responsible for drug-resistance in leukemia. We are the first to demonstrate a direct link between stem cell factor SALL4, SP and drug resistance in leukemia.

## Introduction

Leukemic stem cells (LSCs) are cells that can give rise to leukemia in transplant murine models, and therefore have self-renewal property. It is hypothesized that these cells are not targeted under current chemotherapy regimens and therefore may account for drug resistance and leukemia relapse. Identifying genes or signaling pathways involved in self-renewal of LSCs will likely promote the development of more effective treatments for leukemia and other cancers [1]–[4].

Several methods are currently being used to isolate and study hematopoietic stem cells (HSCs) or LSCs, including the use of HSCs or LSCs cell surface markers [5]–[10] and the use of Hoechst 33342 dye efflux to identify side population (SP) [11]–[16] . There are advantages and disadvantages to using either approach. Isolation of HSCs or LSCs by cell surface markers is better suited for subsequent functional studies such as *in vivo* transplantation since the Hoechst dye is toxic to the cells and therefore might impair their physiological functions. On the other hand, studying SP cells might be a better approach to identify LSCs with potential resistance to chemotherapeutic agents that may account for leukemic recurrence. As characterized by Goodell et al [17], SP from primary AML patients not only has the ability to transfer leukemic phenotypes in NOD-SCID mouse model, but more importantly, these cells have the capacity for rapid efflux of anti-leukemic drugs such as daunorubicin and mitoxantrone. Therefore, SP cells may be a major target for leukemic remission. In addition to blood cells, the SP approach has been used for enrichment for other cancer stem cells, such as breast cancer stem cells [15].

Several ATP binding cassette (ABC) transporter genes have been reported to be responsible for the SP phenotype. Currently, the ABC family has 48 members, which is further divided into 7 subfamilies based on similarities in protein structure, (ABCA, B, C, D, E, F and G) [18] . Among these transporters are the breast cancer resistance protein (BCRP or ABCG2) and ABCA3 [19], [12], [13], [20]–[22]. ABCG2 is one of the earliest ABC transporters to be identified and characterized. It is expressed in normal hematopoietic CD34+ cells and has been associated with a subpopulation of tumor cells that includes tumor stem cells [12], [13], [17]. Later studies have shown that additional ABC transporters are involved in drug resistance of SP cells. ABCA3 is highly expressed in AML patient samples and its expression is associated with unfavorable clinical treatment outcome [23]. Furthermore, the expression of ABCA3 is enriched in leukemic SP cells and has been linked to multidrug resistance by facilitating lysosomal sequestration of drugs in AML primary cells and cell lines [23], [24], [14], [15], [20].

Previously, we have shown that SALL4, a zinc finger transcription factor, forms a core transcriptional network with Oct4, Nanog and Sox2, which governs the self-renewal property of murine embryonic stem (ES) cells [25], [26]. In addition, we found that during normal hematopoiesis, SALL4 is preferentially expressed in human CD34+ hematopoietic stem/progenitors (HS/PCs) and down-regulated in CD34- cells during hematopoietic differentiation [27]. Furthermore, SALL4 is aberrantly expressed in human acute myeloid leukemia, and transgenic SALL4 mice develop acute myeloid leukemia [27]. Loss-of-function studies have demonstrated that SALL4 is a key regulator in leukemic cell survival and down-regulation of SALL4 led to significant apoptosis of leukemic cells [28]. The important role of SALL4 in normal HSC and leukemic stem or initiating cells is supported by its interactions with several key players in self-renewal of HSCs and LSCs: Wnt/β-catenin [27], Bmi-1 [28], [29], and PTEN [30].

In this study, we reported that SALL4 expression was higher in drug resistant primary acute myeloid leukemic patients than those from drug-responsive cases. In addition, while overexpression of SALL4 led to drug resistance in cell lines, cells with decreased SALL4 expression were more sensitive to drug treatments than the parental cells. Having observed that SALL4 was enriched in HS/PCs that were identified by cell surface markers, we planned to evaluate the expression of this stem cell factor in SP and non-SP cells in various blood cancer cell lines (HL-60, NB4, KG1a and RPMI8226 cells), as well as investigate the role of SALL4 in drug resistance and cancer stem cells as identified by SP studies. We found that SALL4 expression was enriched in the SP when compared to the non-SP counterpart. We also found that SALL4 could promote the expression of the ABC transporter genes, such as ABCA3 and ABCG2, and knocking down of SALL4 expression led to a decreased SP. These observations suggest that SALL4 can contribute to the SP phenotype by regulating the expression of ABCA3 and ABCG2. Therefore, by targeting SALL4, we may be able to target the leukemic SP or LSC population.

## Materials and Methods

### Flow cytometry analyses

For side population studies, the cells (1×10^6^/ml) were incubated at 37°C for 90 min with 5 µg/ml Hoechst 33342 (Invitrogen), either alone or in the presence of 50 µM verapamil (Sigma) as a negative control. Side population cells were analyzed on a flow cytometer equipped with 424/444 nm band pass, 670 nm long pass and optical filter. Dead cells were excluded by gating on forward and side scatter which eliminated the PI-positive population. The data were analyzed by FlowJo (Ashland, OR, USA). Freshly sorted SP cells were counted, plated in six-well plates, and cultured with RPMI1640 for 7–8 days. Expanded SP cells were transduced with retrovirus for knocking down of SALL4 and reanalyzed for side population as described below.

### Cell lines and cell culture

All cell cultures were maintained at 37°C with 5% CO_2_. Several human malignant hematopoietic cell lines, NB4, HL-60, KG1a, RPMI8226, 293(embroyonic kidney), MCF7 (breast cancer) and HeLa cell lines were obtained from American Type Culture Collection Inc (ATCC, Lawrenceville, GA, USA).

### Human CD34+ cells

CD34+ cells were purified from primary human blood samples obtained from Lonza Walkersville Inc (Maryland, USA). Briefly, the samples were spun down at 2,400 rpm for 5 seconds at 4°C and the supernatant was discarded, the pellet was then resuspended in 1 mL of 1×PBS supplemented with protease inhibitor cocktail. The cells were then sorted using the EasySep Human Whole Blood/Buffy Coat CD34 Positive Selection Kit (Stemcell Technologies, Vancouver, Canada). 200 µL of the EasySep CD34 antibody serum was added to the sample and incubated at room temperature for 15 minutes, then 100 µL of the EasySep magnetic bead mixture was added and incubated at room temperature for 10 minutes. 3 mL of the sample was selected in a 12×75 mm round-bottom polystyrene tube (BD Falcon, San Jose, CA) using “The Big Easy” EasySep Magnet (Stemcell Technologies). Selection occurred when the sample in the tube was incubated in the magnet at room temperature for 5 minutes, and then the supernatant was discarded. The remaining sample was washed three times with 1.5 mL of 1×PBS supplemented with 2% FBS and 1 mM EDTA, each with a 5 minute incubation period in the magnet between discarding of the supernatant.

### ChIP (Chromatin immunoprecipitation)

293 cells were transiently transfected either with a full length SALL4A, SALL4B or an empty vector. Cells were cross-linked, lysed, and sonicated. Chromatin immunoprecipitations were conducted with a polyclonal anti-SALL4 antibody as described before [28], [29], followed by quantitative real-time PCR. Three sets of ABCA3 and two sets of ABCG2 primers were used to validate the pulldown DNA fragments. Fold enrichment was calculated after normalization with input (no antibody added) of transfected cells. The ABCG2 primers for PCR were 5′- CCTGGATGTCCGGGTCTC-3′ (forward-1), 5′-AACCTTTTGAGTGGGCACAG-3′ (reverse-1), 5′-CTGTGGAGGAACTGGGTAGG-3′ (forward-2), 5-CAAAGGCTCAGGATCTCAGG-3′ (reverse-2) and for ABCA3 was 5′-TCTGGGACGAAGCAGAGAAT-3′ (Positive forward), and 5′- TGAGCATACAGGGGGAAAA-3′ (Positive reverse). 5′-TCTGAGTCAGCCAGTTCTGGTA-3′(Negative1-forward), 5′-CACCCGGTCATATCCTCAGT-3′(Negative1-reverse), 5′-TTTGGCATTTATGTGTCAGGT-3′(Negative2-forward), 5′-TGAGTCTCAATTTCCTATCCCTAAA-3′(Negative2-reverse).

### Quantitative real-time PCR (qPCR)

Pulldown DNA from ChIP was measured by UV spectrophotometry. About 60 ng of DNA was utilized to perform qPCR with iTaq SYBR Green Supermix (Bio-Rad). Reactions were carried out in duplicates.

### Quantitative real-time RT-PCR (qRT-PCR)

Total RNA was extracted with Trizol reagent (Invitrogen) or RNeasy Micro Kit (Qiagen) according to manufacturer's instructions, and the concentration was measured by UV spectrophotometry. 100 ng of total RNA was applied to conduct qRT-PCR by using iScript One-Step RT-PCR Kit with SYBR Green (Bio-Rad). The average threshold cycle for each gene was determined from duplicate reactions and the expression level was normalized to glyceraldehyde 3-phosphate dehydrogenase (GAPDH). SALL4 primers (amplicon length, 68 bp) were the following: 5′- TGCAGCAGTTGGTGGAGAAC -3′ (forward); 5′-TCGGTGGCAAATGAGACATTC -3′ (reverse); and GAPDH primers (amplicon length, 120 bp): 5′- TGTACGCCAACACAGTGCTG -3′ (forward); 5′- TCAGGAGGAGCAATGATCTTG -3 (reverse). ABCA3: 5′-CTCCGAGAAGGACTTTGAGG-3′ (forward); 5′-TCCGTGTGTAACTGAACCGT-3′ (reverse), and ABCG2; 5′-CCCAGGCCTCTATAGCTCAG-3′ (forward); 5′-CGTCAGGAAGAAGAGAACCC-3′ (reverse). The expression levels of SALL4 in AML patients were analyzed by 2-ΔΔCt relative quantitative method.

### Retrovirus production and SALL4 knock-down

The specificity of the SALL4 shRNA has been described in our previous publications [28], [31]. Briefly, three short-hairpin RNA-expressing plasmids, one control (GFP-pRS) and two SALL4 specific (no. 7410, no. 7412; all 3 from Origene, Rockville, MD), were transfected into Phoenix packaging cells (Orbigen, San Diego, CA) using Lipofectamine 2000 (Invitrogen, Frederick, MD). Virus was harvested 48 hours after transfection and filtered through a 0.45-µm filter. Virus supernatant was added to KG1a or CD34+ cells with 8 µg/ml polybrene. Cells were centrifuged at 250× g for 2 hr and incubated for 90 min. After 90 min of incubation, virus supernatant was replaced with fresh medium and incubated for 24 hr.

### MTT assay and drug treatment

2000 cells were seeded into 96-well plates in RPMI 1640 containing 10% FBS. After 20–24 hr, cells were replenished with fresh complete medium containing either a test compound or H_2_O as a vehicle. At the indicated time, the cell proliferation reagent MTS-1 (Promega) was added to each well. The assay was measured at 450 nm using an ELISA Reader (Millipore).

### Patient Study Population

#### Ethics Statement

Discarded peripheral blood samples were obtained from 68 AML patients (AML M2: 42; AML M3: 12; AML M4: 5, AML unclassified: 9) and 30 healthy individuals, between December 2007 and May 2008, in the Peking Union Medical College Hospital, under an approved protocol from the IRB of Peking Union Medical College Hospital. Informed consent was not obtained due to the discarded nature of these samples and the data were analyzed anonymously.

The diagnosis was based on standard cytological criteria according to the French–American–British (FAB) classification [32]. Patient diagnosis was sub-classified by morphology and by immunophenotyping by two independent pathologists. Patient age at diagnosis ranged from 18 to 82 years with a median of 44 years. The AML patients were given standard chemotherapy with most of them responding to the treatments. Ages of the healthy individuals ranged from 14 to 65 years with a median of 41 years.

The 461-AML-cohort was downloaded from Gene Expression Omnibus dataset GSE6891. The data was then analyzed using ParteK Genomic Suite (Missouri, MO, USA). The mean intensity of SALL4 was calculated for the dataset, and each sample was then stratified as SALL4 high or SALL4 low according to their SALL4 expression value.

### Statistical Analysis

SPSS software (version 11.0, SPSS Inc.) was used for statistical analysis of SALL4 expression. All results were presented as a median value (quartile 1∼ quartile 3) because the SALL4 expression in leukemia showed a skewed distribution. Using the Mann-Whitney test, we compared the SALL4 expression between AML in different treatment stages and the healthy control, between pre-treatment and post-treatment AML patients. The Pearson correlation coefficient between SALL4 expression and AML treatment and status was determined. In all cases, a P value <0.05 was considered statistically significant. Statistic comparisons of the ABC genes in SALL4 high- or SALL4 low- group were made using student t-test.

### Electrophoretic Mobility Shift Assays (EMSAs)

DNA-protein binding assays were carried out with nuclear extract from SALL4B transfected HeLa cells prepared with Nuclear Extract kit (Active Motif) according to manufacturer's instructions. Synthetic complementary oligonucleotides were 3′-biotinylated using the biotin 3′-end DNA labeling kit (Pierce) according to the manufacturer's instructions. The sequences of the oligonucleotides used are 5′- GCAGAGAATGGGGAAGACATTTTGGATGGGGTGTTGCAG-3′ (#1), 5′- TCTGTGAAGACTGATGGGGAAAGGGGGTTTTTAGGATGTT-3′ (#2) and 5′-AAGTCAGAGCGCCCTCTGCCGGGCATGGGCAGCATGCAGG-3′ (#3) for ABCA3. Binding reactions were carried out for 20 min at room temperature in the presence of 50 ng/µl poly (dI-dC), 0.05% Nonidet P-40, 10 mm EDTA, and 2.5% glycerol in 1× binding buffer (LightShiftTM chemiluminescent EMSA kit, Pierce) using 20 fmol of biotin-end-labeled target DNA and 2 µg of nuclear extract. Unlabeled target DNA (4 pmol) or 2 µl of anti-SALL4 antibody [28] or anti-Tubulin antibody (Sigma) was added per 20 µl of binding reaction as indicated. Assays were loaded onto native 6% polyacrylamide gels pre-electrophoresed for 60 min in 0.5× Tris borate/EDTA and electrophoresed at 100 V before being transferred onto a positively charged nylon membrane (HybondTM-N+) in 0.5× Tris borate/EDTA at 100 V for 30 min. Transferred DNAs were cross-linked to the membrane at 120 mJ/cm2 and detected using horseradish peroxidase-conjugated streptavidin (LightShiftTM chemiluminescent EMSA kit) according to the manufacturer's instructions. Additional method is listed Text S1.

## Results

### SALL4 expression is associated with AML treatment status

We have shown that SALL4 is aberrantly expressed in AML patients [33], [27]. In this study, we sought to investigate whether SALL4 expression level is associated with AML treatment status. Using the optimized qRT-PCR condition that we established earlier [33], [27], [28], we compared SALL4 expression level in AML patients without treatment (N = 36), AML patients in the complete remission (CR) phase (N = 25), AML patients in the partial remission (PR) phase (N = 7), and healthy controls (N = 30). The definition of CR or PR is based on blast count after the completion of standard chemotherapy. The blast count in bone marrow of the patients in AML CR phase was ≤5%, the blast count in bone marrow with the patient in AML PR phase was between >5% and ≤20%.

**Figure 1:**
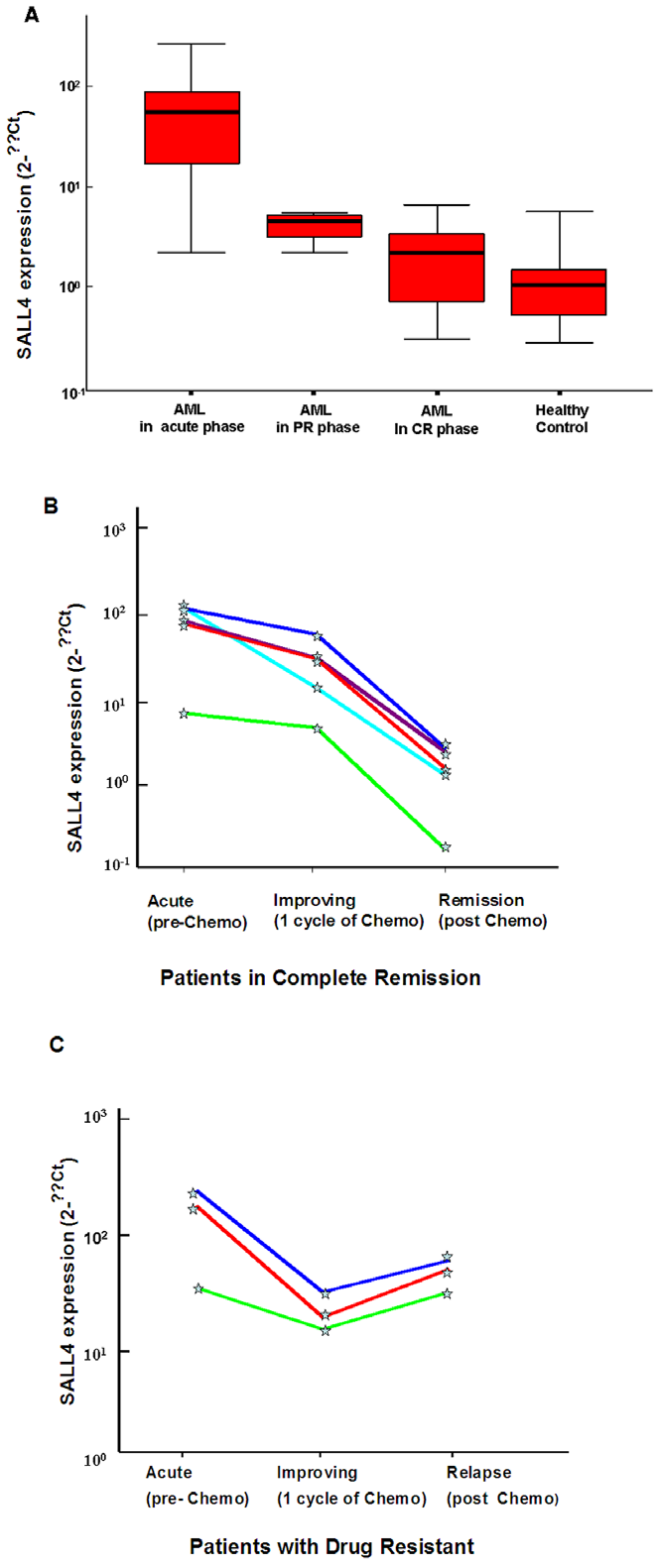
SALL4 expression in AML patients correlated with treatment status. SALL4 had the highest expression level in acute untreated AML patients (N = 36), low expression level in partial remission (PR) patients (N = 7), lower expression level in complete remission (CR) patients (N = 25) and healthy controls (N = 30) (**A**). The level of SALL4 expression declined at remission phase after chemotherapy with decreased blast count in five AML patients in the CR group (**B**, n = 5), but increased in the PR group with drug resistance (**C**, n = 3). Each line represents one patient. Y axis: relative SALL4 expression level reported as 2-^ΔΔ^Ct relative quantitative method.

We noticed that SALL4 had the highest expression level in acute AML patients without treatment [69.01 (range: 17.20∼120.28)], low expression level in PR patients [4.18 (range: 1.76∼5.04)], even lower expression level in CR patients [1.82 (range: 0.50∼2.96)], and the lowest expression level in healthy controls [0. 80 (range: 0.35∼1.13)]. The median level of SALL4 mRNA expression in patients with acute AML without treatment was 86-fold higher than that in the healthy controls (Z = −6.89, p = 0.001), 38-fold higher than that in the AML CR phase (Z = −6.34, p = 0.001), and 17-fold higher than that in the PR phase (Z = −3.61, p = 0.001). The level of SALL4 expression in the AML PR phase was 2-fold higher than that in the AML CR phase (Z = −2.44, p = 0.015, Figure 1A).

Furthermore, we followed up with eight patients to monitor the changes of SALL4 expression during the course of AML treatment at three time points, which included the untreated acute phase, the improving phase (whereby the patient was responding to the chemotherapy as indicated by decreased blast counts, and was at the mid-point of their treatment cycles), and the end of the treatment cycle (of eight patients, five in complete remission (CR) phase, and the other three were in relapse phase). The level of SALL4 expression declined throughout the treatment process for the drug responsive group (Figure 1B) and increased in the drug-resistant group (Figure 1C).

### Overexpression of SALL4 is associated with drug resistance in cell lines

To assess whether the increased expression of SALL4 in AML relapse patients is the consequence or the cause of drug resistance in AML, we compared the proliferation state of a stable cell line with SALL4 overexpression to that of the controls after exposure to two clinically common active chemotherapy agents: doxorubicin and daunorubicin.

**Figure 2:**
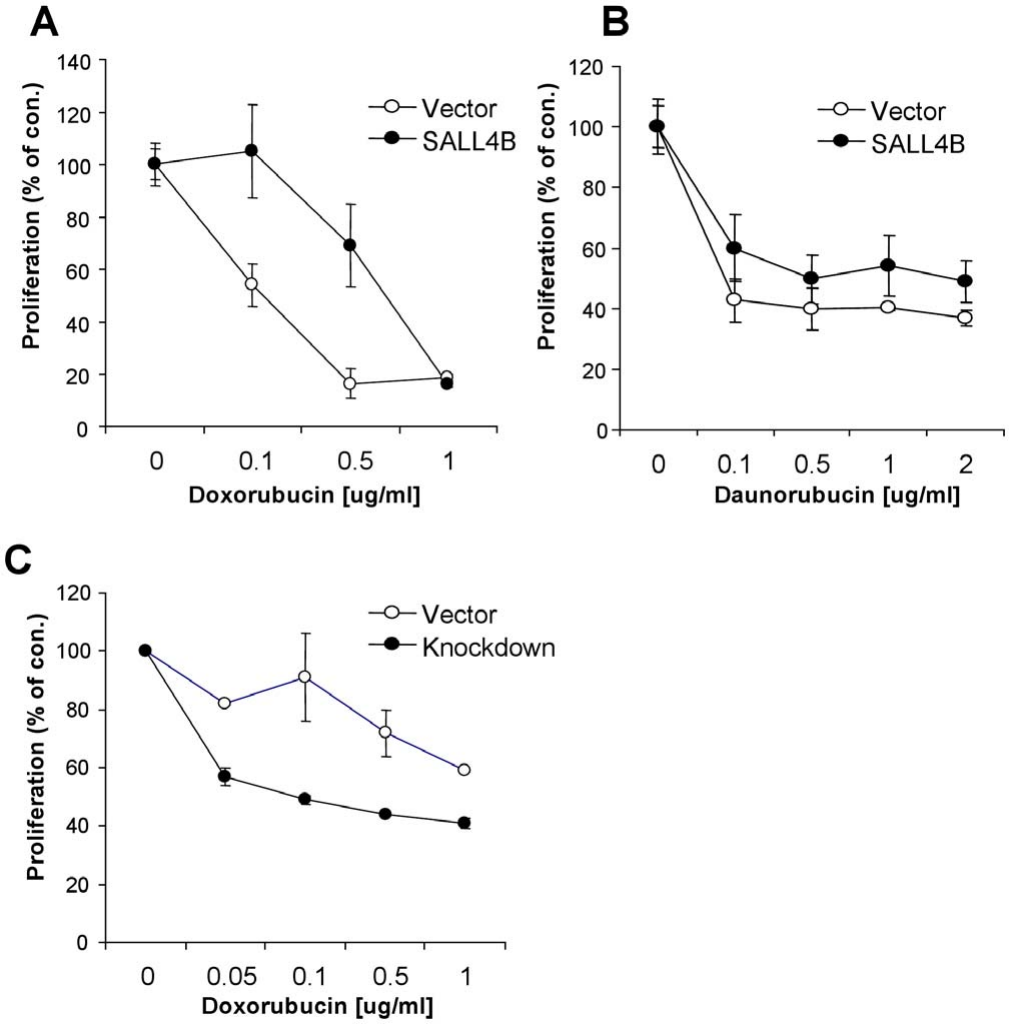
Effects of SALL4 on drug sensitivity. Overexpression of SALL4 resulted in more resistance to doxorubicin (**A**) and daunorubicin (**B**) compared to control cells. 293 cells overexpressing SALL4B (filled circles) or an empty vector (open circles) were seeded in a 96-well plate and cultured with various concentrations of doxorubicin (**A**) or daunorubicin (**B**). At 48 hr after treatment, proliferation was measured with the MTS reagent (Promega). (**C**) Retroviral-mediated SALL4-knockdown KG1a or control cells were treated with doxorubicin. At 24 hr after drug treatment, cell proliferation was measured. Data is represented as mean ± S.D. from three independent experiments. X axis: relative proliferation rate. Y axis: concentrations of doxorubicin or daunorubicin.

To determine the effect of SALL4 in drug resistance, we choose to use 293 cells for the following reasons. First, since SALL4 is expressed in solid tumors such as germ cell tumors (GCTs), gastric cancer, and breast cancer [34], [35] in addition to AML, we hypothesized that SALL4 can confer a general drug resistant phenotype. Second, the 293 cells have relatively lower SALL4 expression compared to blood cell lines, which is better to demonstrate the gain-of functional study. 293 cells overexpressing SALL4B or vector control were plated in a 96-well plate and treated with various concentrations of doxorubicin or daunorubicin. At 48 hours after drug treatment, cell proliferation was measured using the MTS reagent (Promega). Similar to their clinical activity, both agents significantly inhibited the proliferation of 293 cells. As shown in Figure 2A and B, overexpression of SALL4 resulted in a significant reduction in cytotoxicity observed following exposure to doxorubicin (2.0-fold) and daunorubicin (1.5-fold). We next performed the loss-of-function analysis of SALL4. Knocking down of SALL4 in a myeloid leukemic line, KG1a cells, was achieved by retrovirus-mediated delivery of shRNA against SALL4 as we previously described [28], [31]. qRT-PCR analysis showed that retroviral infection markedly repressed SALL4 expression in KG1a cells by 60%. As shown in Figure 2C, SALL4 knock-down cells displayed more sensitivity to doxorubicin than parental cells. These data suggest that SALL4 plays an important role in drug sensitivity.

### Preferential Expression of SALL4 in the SP cells

**Figure 3:**
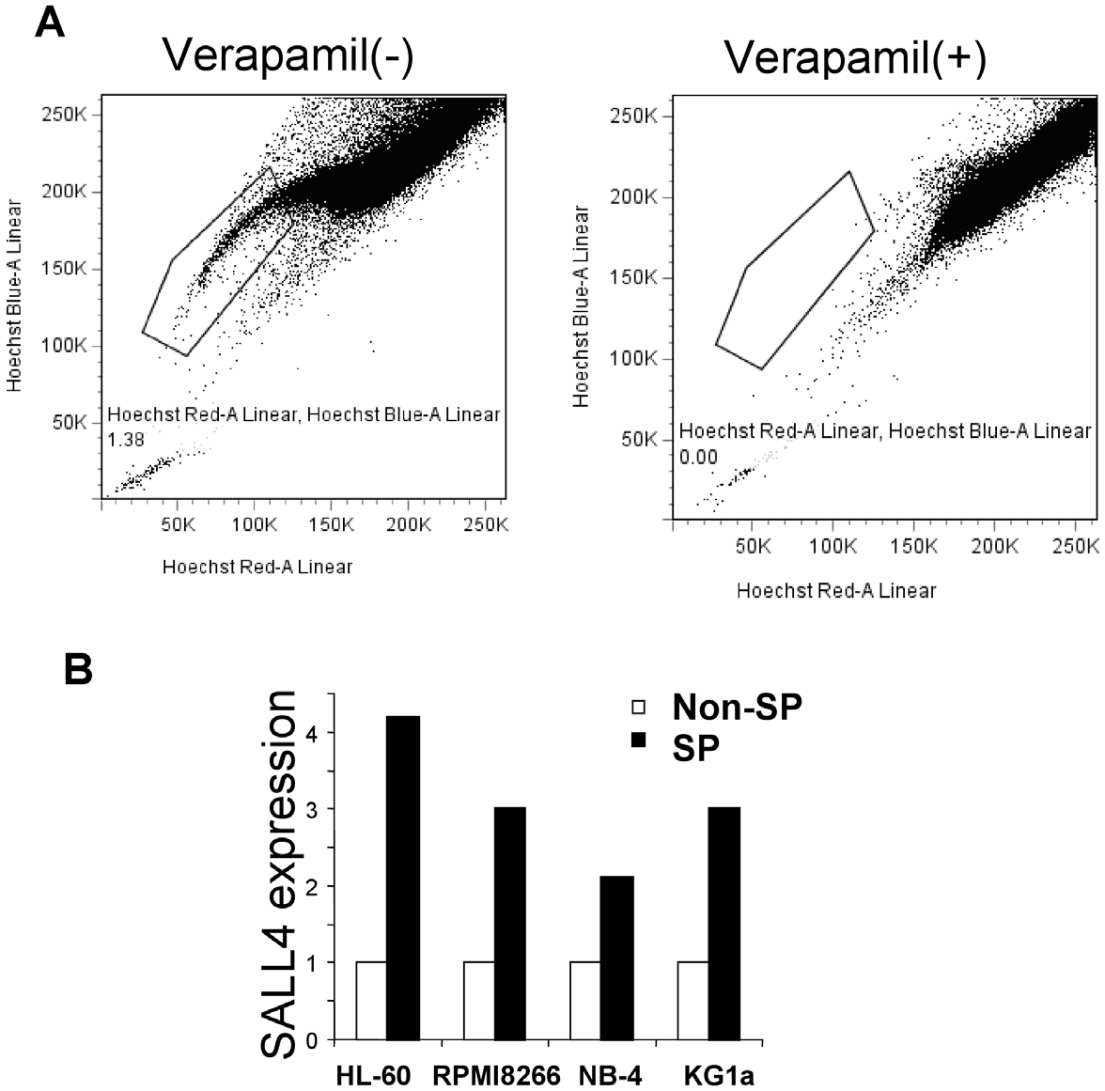
Relative expression of SALL4 in SP and non-SP cells from blood cancer cell lines. (**A**) Representation of FACS profile of SP cells from KG1a. A SP cell profile in the presence of verapamil (50 µM) was at the right as a control. (**B**) qRT-PCR analysis of SALL4 in SP (dark bars) and non-SP (open bars) cells from HL-60, RPMI8226, NB-4 and KG1a cells. For quantification of gene expression, amplification of GAPDH was performed as an endogenous control to standardize the amount of sample. Shown here are the representative results of at least two independent experiments. Y axis: relative SALL4 expression (fold) in comparison to non-SP.

SP cells are defined as cells that show higher efflux of chemicals including DNA-binding dye Hoechst 33342. Therefore, it is not surprising that SP cells are resistant to multiple chemotherapy drugs. Since increased SALL4 expression is associated with drug resistance, we hypothesized that SALL4 might mediate drug resistance at least in part, through the maintenance of SP cells. We first examined the endogenous expression level of SALL4 in SP cells in several hematological cell lines. The SP, as defined by low Hoechst 33342 blue/red fluorescence intensity after incubation with Hoechst 33342, was detected in all cell lines tested at a frequency of 0.5–5%. As a control, this side population disappeared after treatment with verapamil (50 µM) which inhibited the function of the ABC transporter (Figure 3A, right panel). A representative SP profile from KG1a cell line is shown in Figure 3A. We then sorted SP and non-SP cells from four different blood cancer cell lines, HL-60, RPMI8226, NB-4 and KG1a cells, and measured the expression of SALL4 in SP cells and non-SP cells of each cell line by qRT-PCR. Expression of SALL4 was 4.2-, 3.0-, 2.2- and 3.0-fold higher in HL-60, RPMI8226, NB-4 and KG1a SP cells than those from the corresponding non-SP cells, respectively (Figure 3B). Furthermore, SALL4 protein expression was investigated in SP and Non-SP cells by an immunofluorescent stain in KG1a cell line. Consistent with the RNA results, SALL4 was more abundantly expressed in SP cells compared to non-SP KG1a cells (Figure S1). These data suggest that SALL4 is more abundant in SP cells compared to non-SP cells.

### Knocking-down of SALL4 leads to reduced frequency of the side population

To determine if SALL4 is necessary for SP cell maintenance, we next performed loss-of-function study of SALL4 in side population. We first sorted SP cells from KG1a cells. Due to the low number of SP cells, we cultured the sorted SP cells with media containing 10% FBS and expanded them for a week. The SP cells could give rise to both SP and non-SP cells during this culture system, a character that is only observed in SP cells. On the other hand, culture of the non-SP cells yielded no non-SP cells (Figure S2). In addition, *in vivo* transplantation experiments showed that the KG1a SP population had an increased leukemic engraftment when compared to that of the non-SP cells in a xenotransplant mouse model. Four months after injection of 1×10^5^ of SP or non-SP cells into sub-lethally irradiated NOD-SCID mice, the SP recipients had 37% human CD45 cells detected in their bone marrows that were derived from the SP cells, while the non-SP recipients had only 0.1% human CD45 cells (Figure S3). In summary, these features suggest that the SP cells that we sorted from the KG1a cell line are enriched for leukemic stem cell properties and are consistent with other reports. The expanded SP cells were then infected with retroviruses containing shRNA against SALL4 or control scramble shRNA using the same approach as described previously [28]. The SP cell quantification was re-analyzed. qRT-PCR analysis showed that SALL4 expression was reduced by 40%. SALL4 knock-down significantly decreased the percentage of the SP cells from 1.31%to 0.36%. These data suggest that SALL4 plays an important role in the maintenance of side population (Figure S4).

### SALL4 binds to the promoter region of ABCA3 but not ABCG2 gene in vivo

**Figure 4:**
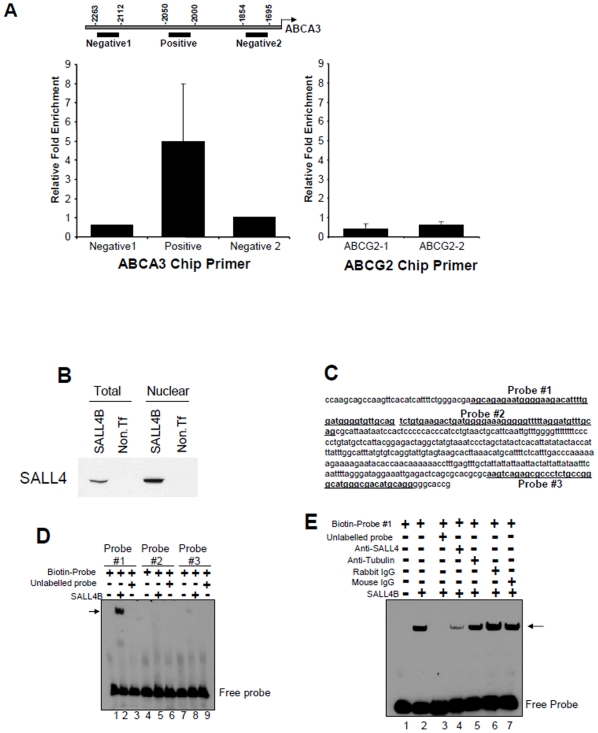
SALL4 binds to the promoter regions of ABCA3. (**A**) ChIP-qPCR showed that SALL4 could bind to ABCA3 but not ABCG2 promoter. Antibody against SALL4 was used to immunoprecipitate DNA fragments from SALL4B-overexpressed 293 cells and fold enrichment was compared to input after normalization with GAPDH. Three pairs of primers in the ABCA3 promoter region (left panel) and two pairs of primers in the ABCG2 promoter region (right panel) were used in ChIP-qPCR analysis. The ABCA3 promoter was found to be bound by SALL4B. (**B**), For EMSA, Western blot analysis using anti-SALL4 antibody was performed on whole cell lysate (left) or nuclear extract (right) from HeLa cells expressing SALL4B. (**C**), EMSA probes sequences (underlined, bold) in the ABCA3 promoter region used for EMSA are indicated. EMSAs were performed to analyze the binding of proteins from nuclear extract of SALL4B expressed HeLa cells to the ABCA3 promoter as described under “Materials and Methods”. (**D**), EMSA using three different probes in the ABCA3 promoter regions. Lane 1–3 are with probe #1, lane 4–6 are with probe #2, and lane 7–9 are with probe #3. Lane 1, 4 and 7 are probe-only reactions, a 200-fold excess of unlabelled probes of each probe was added in lane 3, 6 and 9. (**E**), EMSA was performed with probe #1. Lane 1, labeled oligonucleotide; lane 2, labeled oligonucleotide + nuclear extract; lane 3, labeled oligonucleotide + nuclear extract + 200-fold molar excess of unlabeled oligonucleotides; lane 4, labeled oligonucleotide + nuclear extract + anti-SALL4 antibody; lane 5, labeled oligonucleotide + nuclear extract + anti-Tubulin antibody. Rabbit IgG (lane 6) and mouse IgG (lane 7) were used as controls. Results denote mean ± S.D. from two independent experiments. Y axis: relative fold enrichment of ChIP-promoter region in SALL4B pull-downs when compared to that of input.

It was reported that the ATP binding cassette (ABC) transporters can contribute to drug resistance. We have mapped SALL4 global gene targets using chromatin-immunoprecipitation followed by microarray hybridization (ChIP-on-chip) in myeloid leukemic NB4 cells [28], normal human CD34+ bone marrow cells, and 293 cells (unpublished data). ABCA3, but not ABCG2, was identified as a potential SALL4 downstream target gene in all three cell types. We then sought to validate whether SALL4 could bind to the promoter regions of ABCA3 but not ABCG2 quantitatively using regular ChIP coupled with qPCR. Based on the global chip array results with potential SALL4 binding sites, we designed 3 sets of primers for the ABCA3 promoter region (one was from a peak region, two sets were designed on non-peak regions for negative controls) and two sets of primers for the ABCG2 promoter regions (Figure 4A).

293 cells were transfected with a control vector or SALL4B construct. Total chromatin was isolated and fragmentized by sonication. Chromatin containing SALL4 protein was immunoprecipitated with a SALL4 antibody. To determine whether the promoters of ABCA3 or ABCG2 could be co-immunoprecipitated by the SALL4 complex, we performed PCR reactions to amplify the promoter regions. A fragment of the ABCA3 promoter region was detected in the SALL4 complex, in contrast, neither of the two ABCG2 promoter regions was detected (Figure 4A). Similar data was obtained using KG1a cells ((Figure S5). These data suggest that SALL4 can bind to the ABCA3 promoter directly but probably not to the ABCG2 promoter.

To determine a specific protein-DNA interaction between SALL4 and the ABCA3 promoter, nuclear extracts from HeLa cells overexpressing SALL4B (Figure 4B) were used in EMSA assay. Based on our ChIP-qPCR result, we designed 3 probes (each 40 bp) from ABCA3 promoter region (Figure 4C) and determined which probe could form complex with SALL4B (Figure 4D). As shown in Figure 4D, probe #1 formed a strong complex with SALL4B nuclear extract (lane 2), and the binding was completely ablated with unlabelled oligonucleotide probe #1 (lane 3). In addition, this specific band was ablated after addition of a SALL4 antibody (Figure 4E, lane 4), but not with addition of a Tubulin antibody (Figure 4E, lane 5), IgG (Figure 4E, lane 6 and 7) was used as a negative control. The ablation of a specific band has been reported when an antibody being added to a protein-DNA interaction in an EMSA experiment, and is due to fact that certain antibodies can compete or interrupt the protein-DNA interaction. Though no supershift band was observed with our SALL4 antibody, we interpreted the ablation of a specific band by our SALL4 antibody but not the controls (IgG or Tubulin antibodies) a consequence of competition or interruption of SALL4 protein-DNA interaction by this antibody.

### Transcriptional activation of ABCA3 gene by SALL4

We then sought to investigate whether SALL4 can regulate the ABCA3 promoter by analyzing the human ABCA3 promoter activity. A 5′ upstream of the transcription start site of ABCA3, which contained the SALL4 binding site, was subcloned into the 5′ end of a pGL4.2 basic luciferase reporter plasmid (Figure S6). The SALL4 responsiveness of the ABCA3 promoter was then evaluated through cotransfection of the ABCA3-luciferase construct and Renilla luciferase plasmid together with increasing ratios of the SALL4A or SALL4B expression constructs relative to the ABCA3 promoter construct. As we increased the molar excess of the SALL4A or B constructs, the ABCA3 promoter was activated in a dose-dependent manner (Figure S6).

### SALL4 is able to affect the levels of endogenous expressions of ABCA3 and ABCG2

**Figure 5:**
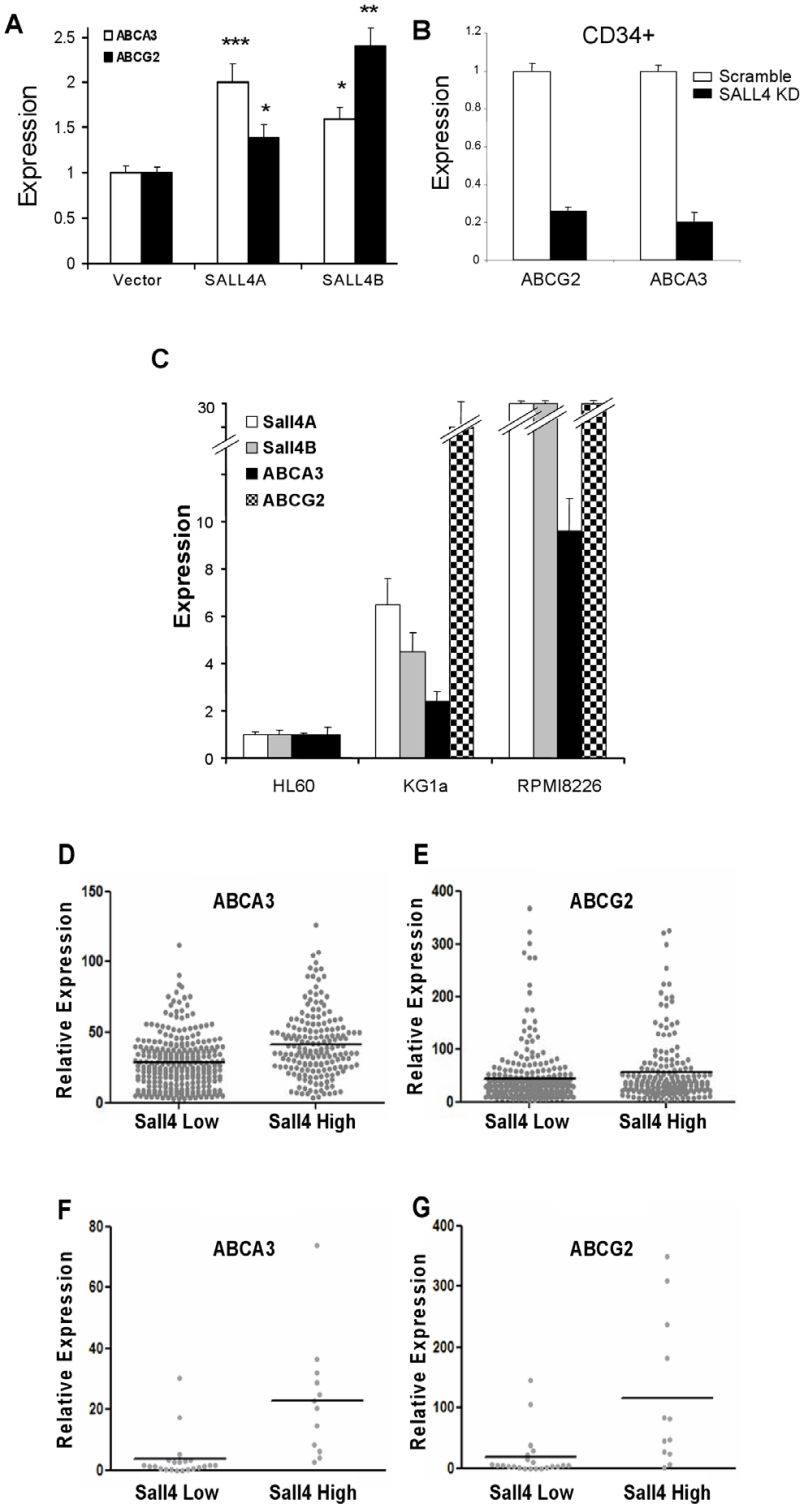
SALL4 affects the levels of endogenous expression of ABCA3 and ABCG2. (**A**) 293 cells were transiently transfected with SALL4A or SALL4B or pcDNA as a control. At 48 hr after transfection, RNA was isolated from transfected cells. Transfected cells were subjected to qRT-PCR to measure the mRNA expressions of ABCA3 and ABCG2. The mRNA expressions of ABCA3 and ABCG2 were significantly increased by SALL4. Data are represented as mean ± S.D. from three independent experiments. *** denotes p<0.001, ** p<0.01, *p<0.05. (**B**) CD34+ cells were transduced with retrovirus expressing shRNA against SALL4 and subjected to qRT-PCR analysis to measure the expression levels of ABCA3 and ABCG2. The expression level of ABCA3 was reduced by 80% and the expression level of ABCG2 was reduced by 74% in CD34+ cells compared to those of the controls. The mRNA level was normalized with the internal control GAPDH. (**C**) qRT-PCR analysis of SALL4A and B, ABCG2, and ABCA3 in HL-60, KG1a, and RPMI8226 cells. For quantification of gene expression, amplification of GAPDH was performed as an endogenous control to standardize the amount of sample. (**D and E**) SALL4 and ABC gene expressions were correlated in primary AML samples. 461 primary AML samples (GSE6891) were stratified into SALL4 high and SALL4 low using the mean intensity of SALL4 as a threshold. (**F and G**) qRT-PCR was performed on 34 primary samples with primers for SALL4, ABCG2 and ABCA3. Mean value of SALL4 was used to stratify the samples to SALL4 high or SALL4 low groups. Data are represented as mean ± S.D. from three independent experiments. Y axis: Relative SALL4 expression when compared to controls.

To determine the regulation of ABCG2 and ABCA3 genes by SALL4, we next examined the expression levels of these two genes in 293 cells overexpressing SALL4A and SALL4B. 293 cells were transiently transfected with SALL4A or SALL4B or pcDNA as a control. At 48 hr after transfection, RNA was isolated from transfected cells. By qRT-PCR, we found that the expression level of ABCA3 in SALL4 overexpressing cells was increased by 33% compared to those of the controls (Figure 5A). Surprisingly, the expression of ABCG2 was increased even more, up to 74%.

We next measured the expressions of ABCA3 and ABCG2 in SALL4-knockdown cells. qRT-PCR analysis showed that retroviral infection with shRNA for SALL4 markedly repressed SALL4 expression in CD34+ cells by 70% (Figure S7). Down-regulation of SALL4 repressed the expression of ABCA3 gene by 80% in CD34+ cells, and similarly, decreased expression level of ABCG2 was observed as well. Similar results were observed for KG1a cells (Figure 5B and data not shown).

We further investigated the correlation of SALL4 and ABCA4 and ABCG2 expression in blood cell lines. SALL4 expression in HL60 was relatively lower than that in KG1a and RPMI8226. Similar to the expression of SALL4, the expression of ABCA3 and ABCG2 was lower in HL60 and higher in KG1a and RPMI8226 cell lines (Figure 5C).

Having established the correlation of SALL4 and the ABC genes in cell lines and CD34+ cells, we next investigated its clinical association in primary AML patients. Using the AML cohort first described by Roel et al [36], we stratified these 461 AML samples into SALL4-high and SALL4-low, according to mean intensity of SALL4. The expression of ABCA3 and ABCG2 was then examined in these two groups. Interestingly, the expression of both genes was significantly higher in the SALL4 high group (P = 0.0001, Figure 5D, and P = 0.02 Figure 5E respectively) compared to SALL4 low group, showing a positive correlation of ABCA3 and ABCG2 with SALL4 in primary AML samples.

To validate, we performed real-time PCR on 34 primary samples from a separate cohort. These samples were stratified with the mean value of SALL4 to SALL4 high and SALL4 low groups. It was found that ABCG and ABCA3 are significantly higher in the SALL4 high population (P = 0.001, P = 0.0001 respectively), and this correlates well with the Roel data set (Figure 5F and G).

Taken together, these results suggest that ABCA3 is a direct downstream target gene of SALL4, and the expressions of two ABC genes, ABCA3 and ABCG2, are positively regulated by SALL4.

## Discussion

The concept of cancer stem/initiating cells is designed to define a cancer subpopulation that has the potential to propagate the tumor and may be responsible for driving tumor development, progression, metastasis and drug resistance or relapse. Xenograft transplantations have been used to test the tumorigenic ability of subsets of cancer cells in immune-compromised mice. It was initially observed that only a very small percentage of tumor cells with unique surface markers had this property, and the bulk of the cells in the tumor lacked this capacity. With the availability of more immune-compromised mouse strains, the spectrum of cancer cells that has this tumorigenic ability has grown broader. In addition, the drug resistance or relapse properties of these cancer initiating cells have not been vigorously tested in this transplantation model using cells isolated by surface markers. Parallel to this approach, the ability of a subgroup of cells to efflux lipophilic fluorescent Hoechst dyes to produce a characteristic side population (SP) profile based on fluorescence-activated flow cytometric analysis has proven valuable as a marker to identify multipotent stem cells in a variety of tissues, including ES cells and various adult stem cells and cancer stem/initiation cells. Therefore, it is more reasonable to combine the transplantation and side population assays to define the cancer initiating cells. The best demonstrated tumor model is from the SP from acute myeloid leukemia samples, which have been shown to have leukemogenic properties in a xenotransplantation model, as well as being resistant to drug treatment [12], [17]. These data indicate that the ability of malignant SP cells to expel anticancer drugs may directly improve their survival and sustain their clonogenicity during exposure to cytostatic drugs, allowing disease recurrence when therapy is withdrawn.

The expressions of several ATP binding cassette (ABC) transporters, including breast cancer resistance protein (BCRP or ABCG2) and ABCA3, are thought to be responsible for expulsion or sequestration of the cytotoxic drugs [23], [24], [12], [13]. These genes are highly expressed in normal hematopoietic CD34+ stem/progenitor cells and SP cells in AML samples [12], [13]. The mechanism(s) involved in the regulation of the expressions of these genes remain under investigation.

SALL4 is a newly discovered pluripotency stem cell factor in murine ES cells. Our group has also shown that it is involved in the self-renewal of leukemic initiation and hematopoietic stem/progenitor cells (HSCs). It is a unique gene that has important functional role(s) in maintaining all three types of stem cells, such as ES cells, HSCs and LSCs. We have shown that SALL4 plays an essential role in myeloid leukemogenesis. It is aberrantly expressed in human primary acute myeloid leukemia [27]. Overexpression of SALL4 in a murine model leads to myeloid leukemic development. Moreover, down-regulation of SALL4 in leukemic cell lines triggers apoptosis [28].

In this study, we correlated the expression of SALL4 with primary AML patient diagnosis and treatment status. We found that AML patients who responded to the treatment had decreasing SALL4 expression throughout the treatment course; while AML patients with disease relapse or drug resistance had increasing SALL4 expression that was correlated to the disease progression. We furthered our study by investigating whether change of SALL4 expression affect chemoresistance (drug responses) of leukemic cells. We found that increased SALL4 expression in leukemic KG1a and 293 cells was associated with chemoresistance, and conversely, knocking down of SALL4 led to a more sensitive response to drug treatment. We then propose that SALL4, a stem cell factor gene, is responsible for the SP phenotype and drug resistance by regulating the ABC genes.

The expression of SALL4 in SP and non-SP cells was investigated and we found that SALL4 was enriched in SP leukemic cells. The preferential expression of SALL4 in SP cells is not limited to the hematopoietic system. In addition to AML, SALL4 has been reported to be expressed in solid tumors such as germ cell tumors (GCTs), gastric cancer, and breast cancer [34], [35]. We have also observed the expression of SALL4 in breast cancer as reported. Moreover, SALL4 is enriched in SP in breast cancer cell line MCF7 along with ABCA3 and ABCG2, and increased SALL4 expression led to an expansion of SP in MCF7 cells (Figure S8–10).

As part of a global SALL4 gene target investigation, we performed ChIP-on-chip on the leukemic cell line NB4, 293 cells, as well as primary CD34+ cells. In all three data sets, we found that SALL4 had potential binding sites for one ABC gene, ABCA3. We further demonstrated that SALL4 could bind to and activate the promoter of ABCA3. Therefore, ABCA3 is a direct target of SALL4. Interestingly, SALL4 could affect the expression levels of both ABCA3 and ABCG2. While overexpression of SALL4 led to increased ABCA3 and ABCG2 expression, knocking down of SALL4 in CD34+ cells and KG1a resulted in decreased expression of ABCA3 and ABCG2, as well as the frequency of SP cells. Most importantly, the expression of SALL4 was positively correlated to those of ABCG2 and ABCA3 in primary leukemic patient samples.

Though there is no evidence to support that ABCG2 is a direct target of SALL4, at least by our study of ChIP-qPCR around its promoter region, it is possible that SALL4 can regulate the expression of ABCG2 through some distal regions which were not investigated by our current study. Alternatively, we have shown that SALL4 represses PTEN expression through recruiting the NuRD complex [30]. The PTEN and Akt pathway has recently been implicated in maintaining the SP phenotype in glioma stem cells and breast cancer stem cells [37], [38], probably through affecting the activity of ABCG2. It is possible that SALL4 affects the expression of ABCG2 (BCRP) indirectly through its regulation of PTEN.

In summary, we are the first to connect a stem cell factor with ABC genes that are responsible for the side population property, a property shared by various normal and cancer stem cells. While the biological implication of SALL4 and ABC transporters in ES cells and other tissue stem cells remain a great interest for further investigation, in this study, we focused on the functional role of SALL4 in the SP of leukemic cells. The side population has been proposed to be a tumor population with intrinsic mechanisms for cytostatic drug resistance that might provide clues for improved therapeutic intervention. Our results suggest that SALL4 plays a role in drug sensitivity, at least in part, through the regulation of ABC genes and maintenance of SP cells, and may be responsible for drug-resistance in leukemia. Further studies will determine whether SALL4 is a novel therapeutic target for leukemic stem/initiation cells in primary AML patients.

## Supporting Information

Figure S1 **SP cells has significantly higher SALL4 protein expression than that of Non-SP cell.** (**A**) Sorted Non-SP cells (upper panel) or SP cells (lower panel) from KG1a cells were immunostained with SALL4 antibody (Green for Sall4, Blue for DAPI). (**B**) SALL4 fluorescence signal per cell was quantified using Image J software.
(TIF)

Figure S2 **Non-SP cells do not give rise to SP cells.** (**A**) KG1a cells were incubated with Hoechst 33342 dye as previously described and analyzed by flow cytometry. Non-SP cells were sorted and cultured with RPMI1640 media for 3 days. (**B**) Cultured Non-SP cells were re-analyzed by flow cytometry and no SP population was identified.
(TIF)

Figure S3 **Increased leukemic engraftment of KG1a SP in vivo in a xenotransplant mouse model.** Four months after injection of 1×10^5^ of SP or non-SP cells into sub-lethally irradiated NOD-SCID mice, the SP recipients had 37% of human CD45 cells detected in their bone marrows which were derived from the SP cells, while the non-SP recipients had only 0.1% of human CD45 cells by FACS analysis. X axis: mouse CD45 expression, Y axis: human CD45 expression.
(TIF)

Figure S4 **Knocking down of SALL4 leads to reduced frequency of the side population.** (**A**) Freshly sorted SP cells from KG1a were counted, plated in six-well plates and cultured with RPMI 1640 for a week. Expanded SP cells were transduced with retrovirus expressing SALL4-specific shRNA for knocking down of SALL4 (lower panel) or scramble shRNA as a control (upper panel) and re-analyzed for side population as previously described. Reduced frequency of the side population was observed in SALL4knockdown cells on the lower pane. (**B**) qRT-PCR analysis showed that SALL4 expression was reduced by 40% in the SALL4 shRNA-treated SP KG1a cells when compared to that of scramble control shRNA treated ones.
(TIF)

Figure S5 **Endogenous SALL4 specifically binds to the ABCA3 promoter revealed by ChIP assay.** Using KG1a cells, ChIP assay was performed by using two antibodies against SALL4 (Sall4-(1): SantaCruz (EE30), (2):in-house antibody) or mouse IgG as a negative control. Enriched chromatin was analyzed by PCR and elctroporesed on agarose gel.
(TIF)

Figure S6 **Activation of the promoter of ABCA3 by SALL4.** (**A**) Diagram of ABCA3-Luc construct which contained the SALL4 binding site. (**B**) ABCA3-Luc construct was cotransfected to HeLa or 293 (data not shown) cells with Renilla luciferase plasmid and increasing ratios of either the SALL4A (gray bars) or SALL4B (black bars) overexpressing constructs. pcDNA (white bars) was used as a control. Data represent the mean of three different experiments. X axis: relative luciferase activity (fold) after overexpression of SALL4A or B in comparison to that of control vector. Y axis: ratio of SALL4A or B construct to control.
(TIF)

Figure S7 **Down-regulation of SALL4 in CD34+ cells.** CD34+ cells were transduced with retrovirus expressing shRNA against SALL4 and subjected to qRT-PCR analysis to measure the expression levels of SALL4 The expression level of SALL4 was reduced by 70% mRNA level was normalized with the internal control GAPDH (N = 3).
(TIF)

Figure S8 **SALL4 is preferentially expressed in SP cells in breast cancer cell line MCF7.** SALL4 protein is expressed in human immature teratoma (II) and breast cancer patients (III and IV). Strong nuclear staining of SALL4 was found in these samples while the negative control (I) with only secondary antibody done on a breast cancer sample showed no nuclear staining at all. Magnification: 200×.
(TIF)

Figure S9 **SALL4 is preferentially expressed in SP cells in breast cancer cell line MCF7.** SP population sorted using the same approach we described for leukemic cell lines and is illustrated in (**A**). SALL4 expression as evaluated by qRT-PCR was over 10 fold enriched in SP cells when compared to non-SP cells. Similar enrichment of expressions of ABCG2 and ABCA3 was also observed in the SP cells from MCF7. Y axis: relative SALL4, or ABCG2, or ABCA3 expression (fold) in comparison to non-SP.
(TIF)

Figure S10 **Increased SALL4 expression led to expansion of SP cells in MCF7 cells.** Overexpression of SALL4B in MCF7 cells increased the SP cells from 0.6% to 3.08% (lower panel), while SP in the control vector treated cells remained unchanged (upper panel).
(TIF)

Text S1 **Supplemental Material.**
(DOC)
