# Supplementary figures and images for: SALL4, a Stem Cell Factor, Affects the Side Population by Regulation of the ATP-Binding Cassette Drug Transport Genes
Source: PLoS One. 2011 Apr 19;6(4):e18372. doi: 10.1371/journal.pone.0018372 (PMC3079717; doi:10.1371/journal.pone.0018372)

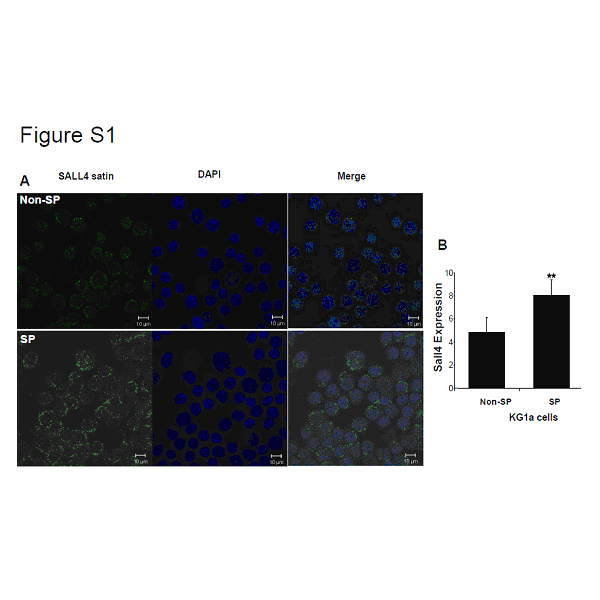

Supplement: Figure S1 — SP cells has significantly higher SALL4 protein expression than that of Non-SP cell. (A) Sorted Non-SP cells (upper panel) or SP cells (lower panel) from KG1a cells were immunostained with SALL4 antibody (Green for Sall4, Blue for DAPI). (B) SALL4 fluorescence signal per cell was quantified using Image J software. (TIF) [file pone.0018372.s001.tif]

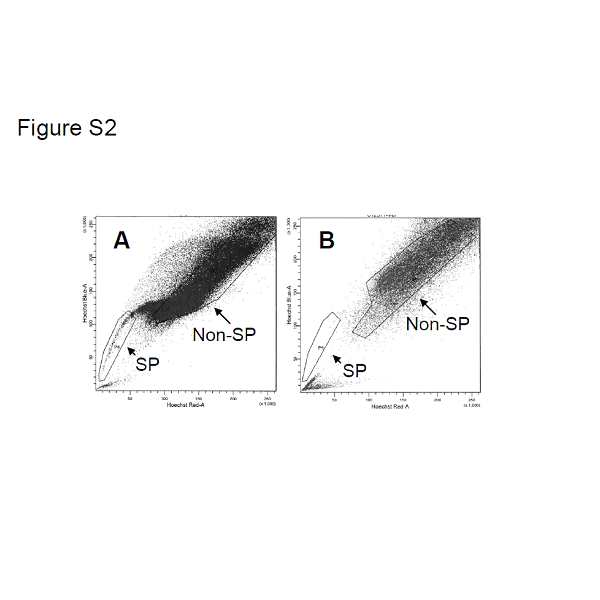

Supplement: Figure S2 — Non-SP cells do not give rise to SP cells. (A) KG1a cells were incubated with Hoechst 33342 dye as previously described and analyzed by flow cytometry. Non-SP cells were sorted and cultured with RPMI1640 media for 3 days. (B) Cultured Non-SP cells were re-analyzed by flow cytometry and no SP population was identified. (TIF) [file pone.0018372.s002.tif]

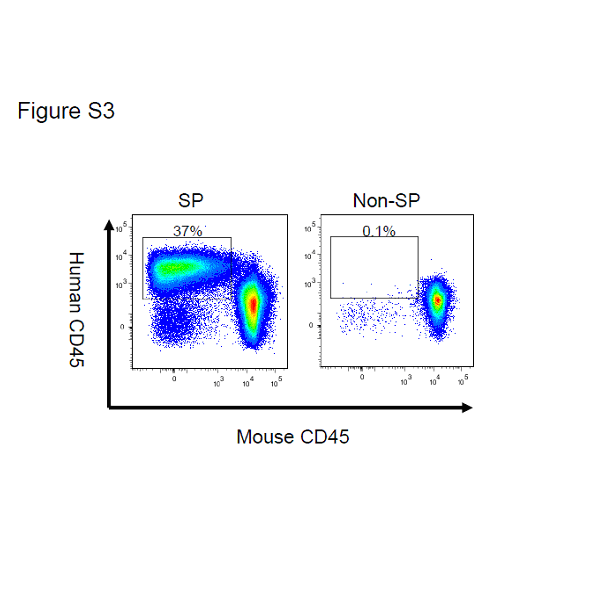

Supplement: Figure S3 — Increased leukemic engraftment of KG1a SP in vivo in a xenotransplant mouse model. Four months after injection of 1×10^5^ of SP or non-SP cells into sub-lethally irradiated NOD-SCID mice, the SP recipients had 37% of human CD45 cells detected in their bone marrows which were derived from the SP cells, while the non-SP recipients had only 0.1% of human CD45 cells by FACS analysis. X axis: mouse CD45 expression, Y axis: human CD45 expression. (TIF) [file pone.0018372.s003.tif]

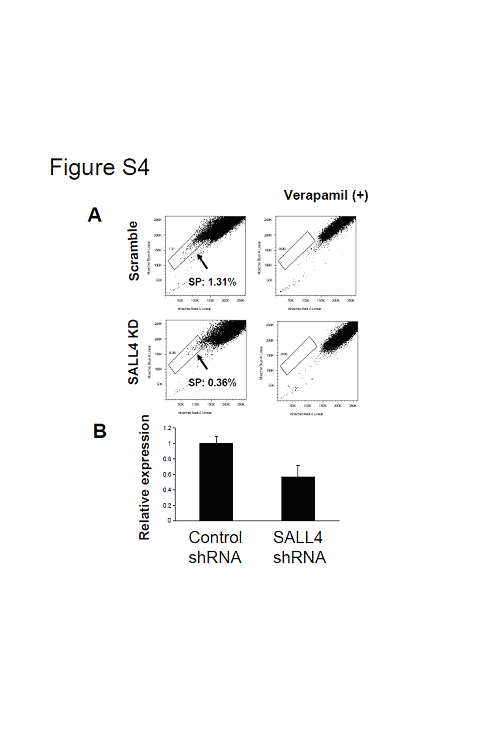

Supplement: Figure S4 — Knocking down of SALL4 leads to reduced frequency of the side population. (A) Freshly sorted SP cells from KG1a were counted, plated in six-well plates and cultured with RPMI 1640 for a week. Expanded SP cells were transduced with retrovirus expressing SALL4-specific shRNA for knocking down of SALL4 (lower panel) or scramble shRNA as a control (upper panel) and re-analyzed for side population as previously described. Reduced frequency of the side population was observed in SALL4knockdown cells on the lower pane. (B) qRT-PCR analysis showed that SALL4 expression was reduced by 40% in the SALL4 shRNA-treated SP KG1a cells when compared to that of scramble control shRNA treated ones. (TIF) [file pone.0018372.s004.tif]

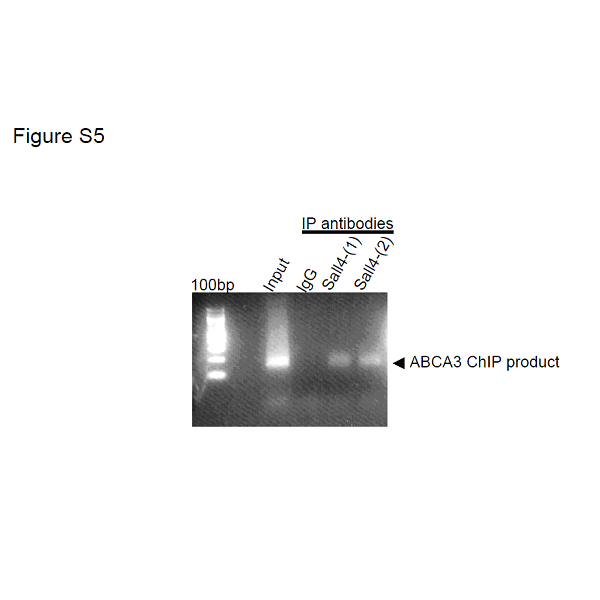

Supplement: Figure S5 — Endogenous SALL4 specifically binds to the ABCA3 promoter revealed by ChIP assay. Using KG1a cells, ChIP assay was performed by using two antibodies against SALL4 (Sall4-(1): SantaCruz (EE30), (2):in-house antibody) or mouse IgG as a negative control. Enriched chromatin was analyzed by PCR and elctroporesed on agarose gel. (TIF) [file pone.0018372.s005.tif]

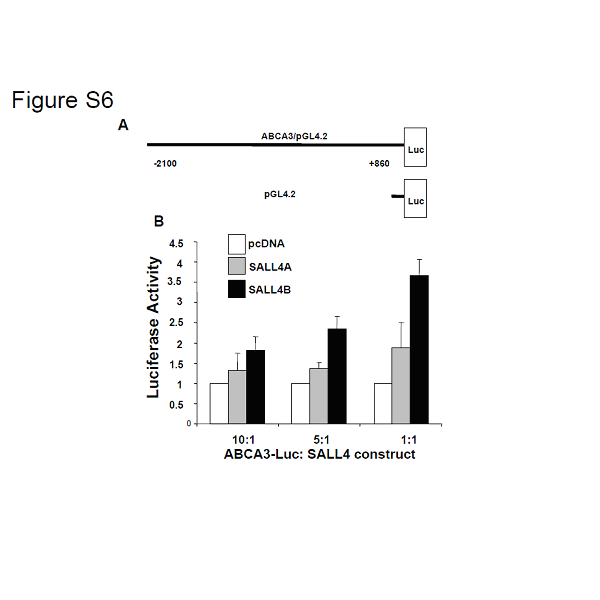

Supplement: Figure S6 — Activation of the promoter of ABCA3 by SALL4. (A) Diagram of ABCA3-Luc construct which contained the SALL4 binding site. (B) ABCA3-Luc construct was cotransfected to HeLa or 293 (data not shown) cells with Renilla luciferase plasmid and increasing ratios of either the SALL4A (gray bars) or SALL4B (black bars) overexpressing constructs. pcDNA (white bars) was used as a control. Data represent the mean of three different experiments. X axis: relative luciferase activity (fold) after overexpression of SALL4A or B in comparison to that of control vector. Y axis: ratio of SALL4A or B construct to control. (TIF) [file pone.0018372.s006.tif]

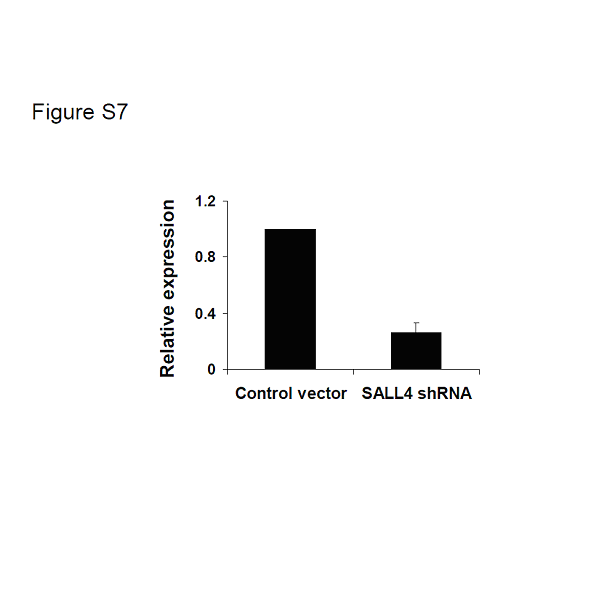

Supplement: Figure S7 — Down-regulation of SALL4 in CD34+ cells. CD34+ cells were transduced with retrovirus expressing shRNA against SALL4 and subjected to qRT-PCR analysis to measure the expression levels of SALL4 The expression level of SALL4 was reduced by 70% mRNA level was normalized with the internal control GAPDH (N = 3). (TIF) [file pone.0018372.s007.tif]

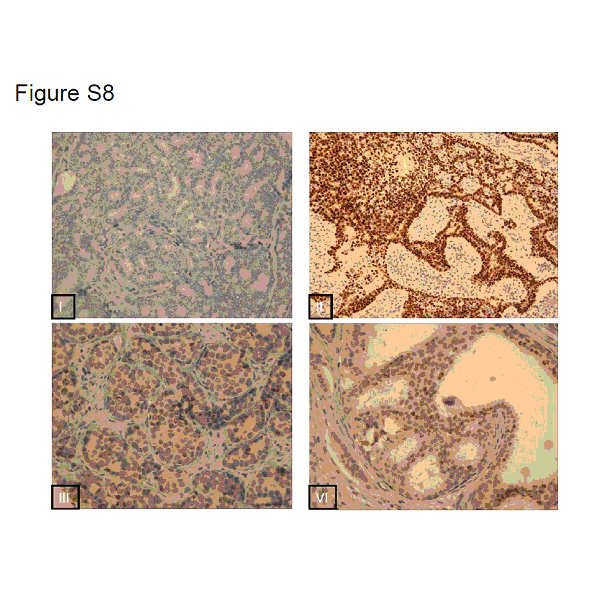

Supplement: Figure S8 — SALL4 is preferentially expressed in SP cells in breast cancer cell line MCF7. SALL4 protein is expressed in human immature teratoma (II) and breast cancer patients (III and IV). Strong nuclear staining of SALL4 was found in these samples while the negative control (I) with only secondary antibody done on a breast cancer sample showed no nuclear staining at all. Magnification: 200×. (TIF) [file pone.0018372.s008.tif]

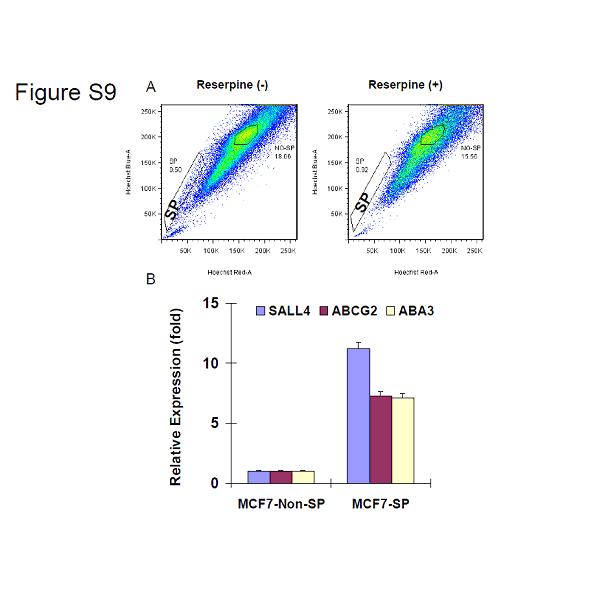

Supplement: Figure S9 — SALL4 is preferentially expressed in SP cells in breast cancer cell line MCF7. SP population sorted using the same approach we described for leukemic cell lines and is illustrated in (A). SALL4 expression as evaluated by qRT-PCR was over 10 fold enriched in SP cells when compared to non-SP cells. Similar enrichment of expressions of ABCG2 and ABCA3 was also observed in the SP cells from MCF7. Y axis: relative SALL4, or ABCG2, or ABCA3 expression (fold) in comparison to non-SP. (TIF) [file pone.0018372.s009.tif]

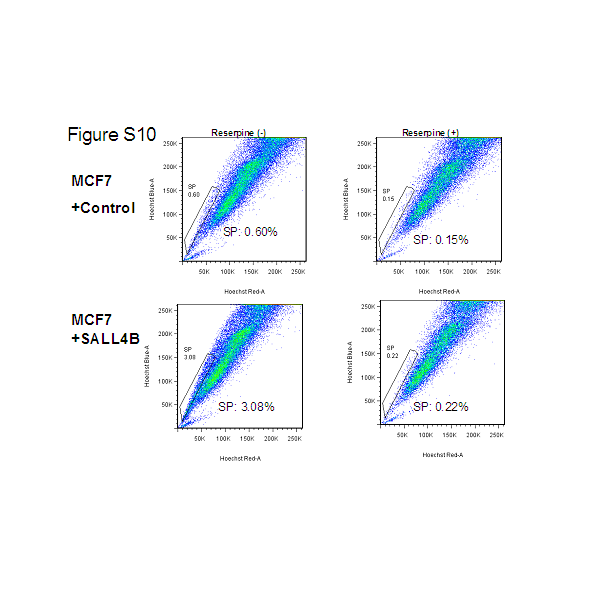

Supplement: Figure S10 — Increased SALL4 expression led to expansion of SP cells in MCF7 cells. Overexpression of SALL4B in MCF7 cells increased the SP cells from 0.6% to 3.08% (lower panel), while SP in the control vector treated cells remained unchanged (upper panel). (TIF) [file pone.0018372.s010.tif]
