# Supplementary material for: SALL4, a Stem Cell Factor, Affects the Side Population by Regulation of the ATP-Binding Cassette Drug Transport Genes
Source: PLoS One. 2011 Apr 19;6(4):e18372. doi: 10.1371/journal.pone.0018372 (PMC3079717; doi:10.1371/journal.pone.0018372)
Supplement: Text S1 — Supplemental Material. (DOC) [file pone.0018372.s011.doc]

**Text S1: Supplemental Material**

**Immunofluorescent staining and confocal imaging**

The SP or Non-SP cells from KG1a cells were sorted and cytospun on slides at 1,000 rpm for 8 min. The collected cells were fixed with 4% PFA for 5 min, blocked with 10% BSA in PBS for 1 hr and were incubated with anti-Sall4 antibody (1:400) overnight at 4C followed by incubation with a secondary antibody (Alexa Flour 488 anti-rabbit IgG (1:1,000) (Invitrogen)) in PBS for 30 min at room temperature. Cells were mounted by using Vectashield mounting medium with 4, 6-diamidino-2-phenylindole (DAPI, Vector Laboratories). All fluorescence images were generated using a Carl Zeiss LSM510 scanning laser confocal microscope. Fluorescence signal per each cell was quantified using Image J software.

**Xenotransplantation**

1 × 10^5^ of SP or non-SP KG1a cells were transplanted into sublethally irradiated (1.0 Gy) NOD/SCID/IL2rγnull mice (NOD.Cg-Prkdcscid Il2rgtm1Wjl/SzJ, The Jackson Laboratory, ME USA) by tail vein injection. Peripheral blood (PB) and bone marrow (BM) chimerism was monitored at 16 weeks post transplantation. Bone marrow cells were obtained for flow cytometry analysis utilizing FITC-conjugated antihuman CD45 antibody and APC-conjugated anti-mouse CD45 antibody (eBiosciences, CA USA). The percentage of human CD45+ cells was calculated as follows: % human CD45+ cells = No. human CD45+ cells/ (No. human CD45+ cells + No. mouse CD45+ cells) x 100.

**Promoter assays**

To examine transcriptional activity of ABCA3 by SALL4, 293 and HeLa cells were grown in six-well plates. Cells were transiently co-transfected with 1 ug of the ABCA3 promoter luciferase reporter construct, SALL4A or B expression construct (pcDNA-SALL4A and B) and Renilla luciferase construct by Fugene 6, according to the manufacturer’s protocol. After 24hr, the cells were harvested and analyzed for luciferase activities using assay kits (Dual-Luciferase Reporter Assay) from Promega. The data are represented as the ratio of firefly to Renilla luciferase activity. Each experiment was performed in triplicate.

**Construction of reporter plasmids**

DNA fragments, nucleotides −2158 to +860 relative to the transcriptional on ABCA3 gene, were prepared by PCR amplification using genomic DNA as a template and digestion with appropriate restriction enzymes. The DNA fragment was inserted upstream of the firefly luciferase gene in the pGL4.2 reporter plasmid (Promega), and was designated as ABCA3-Luc. The nucleotide sequence of each construct was verified by sequencing.
